# Supplementary material for: Predictors of Influenza Vaccination among Chinese Middle School Students Based on the Health Belief Model: A Mixed-Methods Study
Source: Vaccines (Basel). 2022 Oct 26;10(11):1802. doi: 10.3390/vaccines10111802 (PMC9697411; doi:10.3390/vaccines10111802)
Supplement: Supplementary file 1 [file vaccines-10-01802-s001.zip › vaccines-1971158-supplementary.pdf]

The items based on HBM

Dimensions	No	Questions	1	2	3	4	5
Perceived susceptibility	A1	I belong to a susceptible group					
	A2	I had friends and family whoever had flu					
	A3	Influenza vaccination is very important					
Perceived benefits	B1	Influenza vaccine will help us free of influenza					
	B2	Influenza vaccine will help me avoid missing class because of illness					
	B3	Influenza vaccine will help save my parents' time to care for me					
	B4	Influenza vaccine can protect my family from infection					
Perceived barriers	C1	I am worried about adverse reactions after the influenza vaccination					
	C2	I refused to get vaccinated because I was afraid of needles' prick					
	C3	Vaccination may take up my class time					
	C4	Family members will not be present during vaccination					
	C5	The inoculation site is noisy and make me feel uncomfortable.					
	C6	Medical staff are not friendly					
	C7	My classmates would observe me and I would feel embarrassed					
Cues to action	D1	The medical staff told me that the influenza vaccine is good for me and I should be vaccinated					
	D2	According to CDC public service announcement that the influenza vaccine is good for me and I should be vaccinated					
	D3	TV news, WeChat subscriptions and other mass media say that the influenza vaccine is good for me and I should be vaccinated					
Self-efficacy	E1	When I openly discuss vaccination with others, I may positively influence others in their beliefs in vaccination					
	E2	When I openly discuss vaccination with others, they may positively influence me					
	E3	I tend to listen to my parents about vaccinating or not.					

1"strongly disagree"; 2"disagree";3"not sure"; 4"agree"; 5"strongly agree".
